# A review of the New World species of the parasitoid wasp *Iconella* (Hymenoptera, Braconidae, Microgastrinae)

**DOI:** 10.3897/zookeys.321.5160

**Published:** 2013-08-07

**Authors:** José L. Fernández-Triana, Sophie Cardinal, James B. Whitfield, Winnie Hallwachs, M. Alex Smith, Daniel H. Janzenr

**Affiliations:** 1 Biodiversity Institute of Ontario, University of Guelph, Guelph, ON N1G 2W1 Canada; 2 Canadian National Collection of Insects, Agriculture and Agri-Food Canada, 960 Carling Ave., Ottawa, ON, K1A 0C6 Canada; 3 Department of Entomology, University of Illinois, Urbana, IL 61801 USA; 4 Department of Biology, University of Pennsylvania, Philadelphia, PA 19104-6018 USA; 5 Department of Integrative Biology, University of Guelph, Guelph, ON, N1G 2W1 Canada

**Keywords:** *Iconella*, Microgastrinae, New World, taxonomic review, host caterpillars, DNA barcoding, Area de Conservacion Guanacaste, parasitoid wasps

## Abstract

The New World species of *Iconella* (Hymenoptera: Braconidae, Microgastrinae) are revised. *Iconella andydeansi* Fernández-Triana, **sp. n.**, *Iconella canadensis* Fernández-Triana, **sp. n.**, and *Iconella jayjayrodriguezae* Fernández-Triana, **sp. n.**, are described as new. *Iconella isolata* (Muesebeck, 1955), **stat. r.**, previously considered as a subspecies of *Iconella etiellae* (Viereck, 1911), is here elevated to species rank. All species have different, well defined geographic distributions and hosts. Taxonomic keys are presented in two formats: traditional dichotomous hardcopy versions and links to electronic interactive versions (software Lucid 3.5). Numerous illustrations, computer-generated descriptions, distributional information, host records (mostly Lepidoptera: Crambidae and Pyralidae), and DNA barcodes (where available) are presented for every species. Phylogenetic analyses of the barcoding region of COI indicate the possibility that *Iconella* is not monophyletic and that the New World species may not form a monophyletic group; more data is needed to resolve this issue.

## Introduction

The genus *Iconella* was erected by Mason (1981) to accommodate a group of *Apanteles* species with a sinuated vein cu-a in the hind wing, a character he interpreted as plesiomorphic among Microgastrinae. *Iconella* includes all species of Nixon’s *sundanus*-group and part of the *merula*-group (Nixon 1965, Whitfield 1997). It is cosmopolitan, with 33 described species, most of them found in the Palearctic (Yu et al. 2012). All species with known hosts are solitary parasitoids of microlepidoptera, especially concealed feeders (Whitfield 1997); one species, *Iconella isolata* (Muesebeck, 1955) has been extensively studied in the tropics for biological control of a pest caterpillar (e.g. Bennett 1960, Bartlett et al. 1978).

Until now, only one species was known from the New World, *Iconella etiella* (Viereck, 1911). However, ongoing research on the Microgastrinae fauna of Area de Conservacion de Guanacaste (ACG) in northwestern Costa Rica (Smith et al. 2008, Janzen et al. 2009) and study of specimens housed in the Canadian National Collection of Insects (CNC) in Ottawa, Canada, have both revealed several new species for the Western Hemisphere. They are described here, and a key to all species in the New World is provided.

It should be noted that Achterberg (2002) did not consider *Iconella* as a valid genus and transferred its species (as well as those of other genera) back to *Apanteles*. However, that decision has not been universally accepted (e.g. Fernández-Triana 2010, Broad et al. 2012) and is still a subject of debate. In this paper we treat *Iconella* as a valid genus on its own.

## Methods

*Iconella* is rare in collections, and even when present, specimens tend to be categorized as “unidentified Microgastrinae”. In the New World, with the exception of one species, the genus seems to be rare in nature as well. Even long-term and comprehensive rearing programs –such as those in ACG, Costa Rica- have recovered relatively few specimens. This study is mostly based on the examination of unidentified *Iconella* specimens housed in the CNC, representing close to 40 specimens; 11 specimens reared by the ACG inventory -and housed in the CNC and the Illinois Natural History Survey, Champaign, Illinois, United States(INHS); one specimen from the Laurentian Forestry Center, Ste.-Foy, Quebec, Canada(LFS); and the holotype of *Iconella etiellae* from the Smithsonian Institution, Washington DC, United States(NMNH).

Morphological terms and measurements of structures are mostly as used by Mason (1981), Huber and Sharkey (1993) and Whitfield (1997). Non-morphological characters are also provided in the key whenever available (e.g., host species, geographical distribution). Those features are included in brackets at the end of the corresponding couplet and are intended as supplementary information that can help with identification.

The species descriptions are based mostly on the holotype female, with other specimens studied (when available) for intraspecific variation. When the holotype could not be examined (*Iconella isolata*) or it was a male (*Iconella etiellae*), female specimens were used for these redescriptions.

Lucid 3.5.4 (http://www.lucidcentral.com/) software was used to generate automatic descriptions of the species and to prepare Lucid identification keys. A dataset of 20 characters and 126 character-states was used to provide uniform descriptions for all species treated. Description format includes one sentence per character, with the character (in italics) mentioned first and the character-state following after a colon, e.g., “*Pterostigma color*: mostly brown, with yellowish-white spot at anterior 0.2 ×”.

A map with the distribution of all New World species of *Iconella* was generated using SimpleMappr (Shorthouse 2010).

In the taxonomic treatment of species, “Specimens Examined” presents the specimen’s information in the following format: “Number of females/males, acronym of the storing institution between parentheses, COUNTRY: State/Province, city, other locality details, coordinates (in Decimal Degrees, abbreviated as “Lat:” and “Long:”), date, collector name, biological information on host (starting with “ex”), ACG database codes (in the format “yy-SRNP-xxxxxx” or “DHJPARxxxxxxx”, with the former being the voucher code of the host and the latter being the voucher code of the parasite). For states of the United States and for Canadian provinces/territories, acronyms consisting of two capital letters are used, following Canada Post (http://www.canadapost.ca/tools/pg/manual/PGaddress-e.asp).

DNA barcodes for all specimens that were barcoded were obtained using DNA extracts prepared from single legs using a glass fibre protocol (Ivanova et al. 2006). Extracts were re-suspended in 40 μl of dH2O, and a 658-bp region near the 5’ terminus of the COI gene was amplified using standard primers (LepF1–LepR1) following established protocols (Smith et al. 2006, 2007, 2008). If the initial 658 bp amplification was not successful, composite sequences were generated using internal primers (primers are as detailed in Smith et al. (2008)). All available DNA barcodes for *Iconella* specimens from the New World and the Palearctic are available on the Barcode of Life Data System (BOLD, www.boldsystems.org/), along with sequences from *Apanteles* and *Dolichogenidea*, which were used as outgroups in the phylogenetic analyses (doi.org/10.5883/DS-ASICON1). Collection information and accessions (BOLD and GenBank) for all sequences were already published in a previous paper (Smith et al. 2013).

DNA barcode sequences were aligned in Geneious Pro 6.0.5 (Drummond et al. 2011) using the default settings for a MUSCLE alignment. Because of the high AT content characteristic of Insect and Hymenoptera mitochondrial DNA, sequence divergences were calculated using the TN93 model (Tamura and Nei 1993) and a neighbor-joining (NJ, Saitou and Nei 1987) tree of distances was generated using Geneious Pro 6.0.5 (Drummond et al. 2011) to provide a graphical representation of the species divergences.

Many of the *Iconella* specimens in BOLD were collected before 1979. Characteristic of such moderately-aged specimens, the COI fragments generated were less (~160bp) than the full length characteristic of the standard DNA barcode (>600bp) (Fernández-Triana et al. 2011). Therefore, more than half of the sequences used in the analyses described below were less than 300bp, and 44% were less than 200bp. Phylogenetic comparisons of fragments this small, and involving cases where there is even lower overlap will be compromised. However, small COI fragments (<200bp) are not devoid of phylogenetic signal and have been used to successfully identify species (Fernández-Triana 2010, Fernández-Triana et al. 2011, Hajibabaei et al. 2006).

To investigate the phylogenetic relationships of the species, the aligned dataset was analyzed in MrBayes v. 3.2.1 (Ronquist and Huelsenbeck 2003). Model selection was based on the Akaike Information Criterion as implemented in JModelTest v.2.1.1 (Darriba et al. 2012). Two independent analyses with 4 chains each were run in parallel for 10 million generations under a GTR+I+G model. The parameter trace files of each run were observed in Tracer v.1.5 (Rambaut and Drummond 2009) to verify that the runs had converged on the same stationary distribution, and to decide on the appropriate number of generations to discard as burn-in. A 50% majority rule consensus tree was constructed from the 18 million post-burn-in generations in Geneious Pro 6.0.5. The above protocol was followed for additional analyses in which 1) all 3^rd^ codon positions were removed from the dataset, 2) characters with more than 2% missing data were removed, and 3) sequences that were less than 547 base pairs long were eliminated. A Maximum Likelihood (ML) analysis was also run in RAXML v. 7.3.4 (Stamatakis 2006) under a GTR+I+G model. 1000 bootstrap (BS) replicates were run and the BS support values were then drawn onto the best-scoring ML tree. A parsimony analysis was conducted in TNT v.1.1 (Goloboff et al. 2008) using the “aquickie.run” script provided with the program.

## Results

As with many other taxa, the generic status of *Iconella* will only be solved when a comprehensive phylogeny of Microgastrinae is carried out. In the meantime we think is best to keep it as a valid genus, based on the available morphological evidence. Mason (1981) already mentioned the sinuated vein cu-a in the hind wing as a plesiomorphic character that suggests the unique status of *Iconella* among similar genera. Besides that, we also consider the presence of a median longitudinal carina on the propodeum (or the secondary loss of that carina, which occurs in some Paleartic species but not in the New World species) as a strong support for the generic status of *Iconella* –in contrast with *Apanteles*, which (*sensu* Mason 1981) almost always has carinae defining a complete or partial areola on the propodeum, but never has a median longitudinal carina on the propodeum.

**Figure 1.**
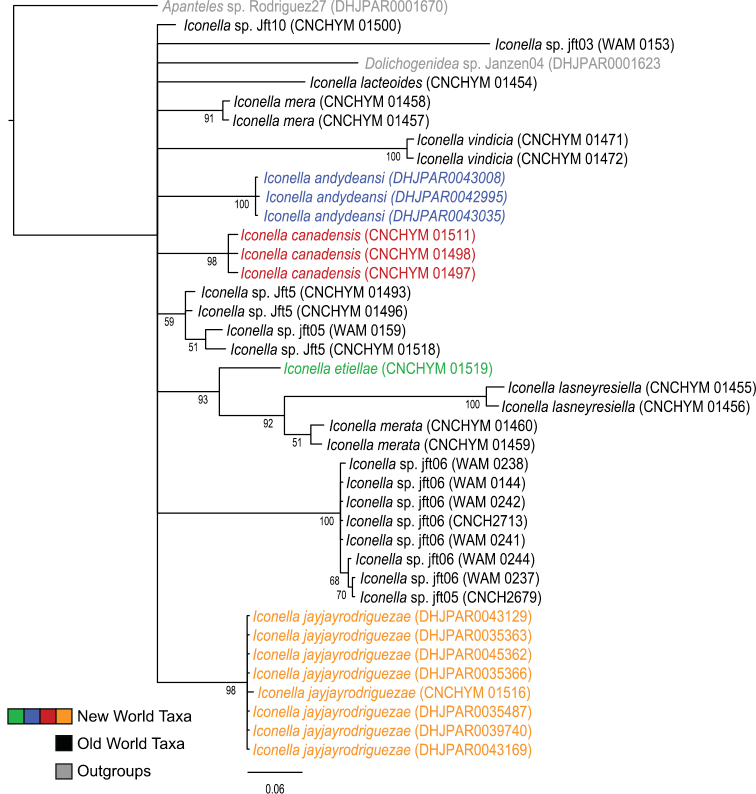
Majority rule consensus tree calculated from the posterior tree samples of the Bayesian analysis of the full *Iconella* COI fragment dataset. Values below the branches are posterior probabilities. The New World species are individually color coded.

**Figure 2.**
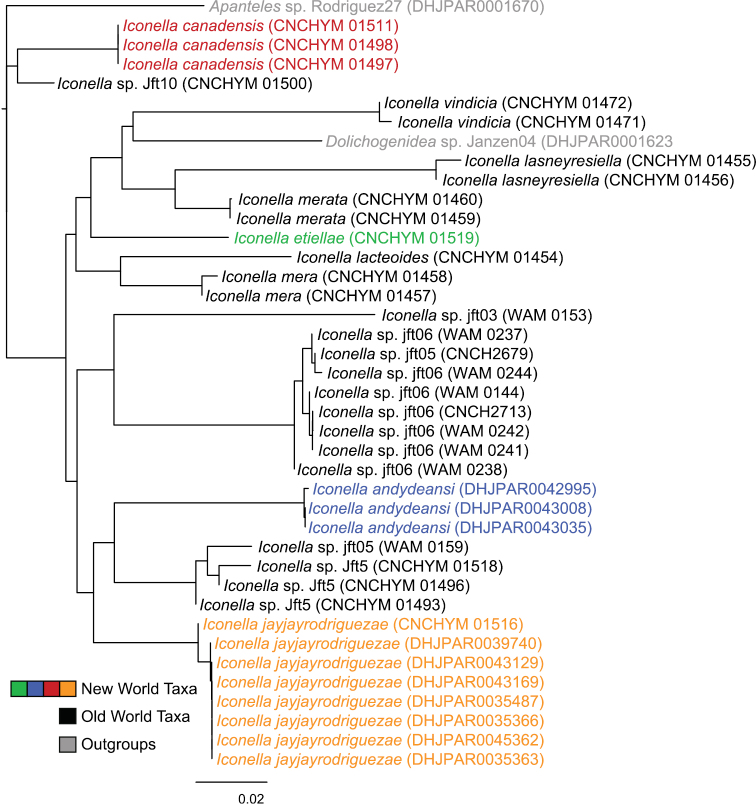
Neighbor-joining tree of the full *Iconella* COI fragment dataset using the TN93 model. The New World species are individually color coded.

Phylogenetic analyses of the COI DNA, however, are inconclusive as to whether the genus *Iconella* is monophyletic (Figs 1 and 2). All Bayesian (only the tree resulting from analysis of the full dataset is shown in Fig. 1), ML (tree not shown), and parsimony (tree not shown) analyses failed to recover monophyly of the genus. However, the model-based methods do not offer strong support against monophyly either. The highest posterior probability (PP) supporting non-monophyly was 0.55 when 3^rd^ codon positions were removed from the Bayesian analysis, and BS support against monophyly in the ML analysis was only 0.21.

We recognize five species of *Iconella* in the New World, including the three new species described in the present paper, and one that was previously considered as a subspecies (Muesebeck 1955) but is here elevated to species. We are also aware of an additional sixth species, represented by two specimens in poor condition from British Columbia (Western Canada), deposited in the CNC. They were mentioned in Fernández-Triana (2010) as “*Iconella* sp. 2” and are different from all other New World species of the genus. However, until more material is found, it is impossible to describe that species.

**Figure 3.**
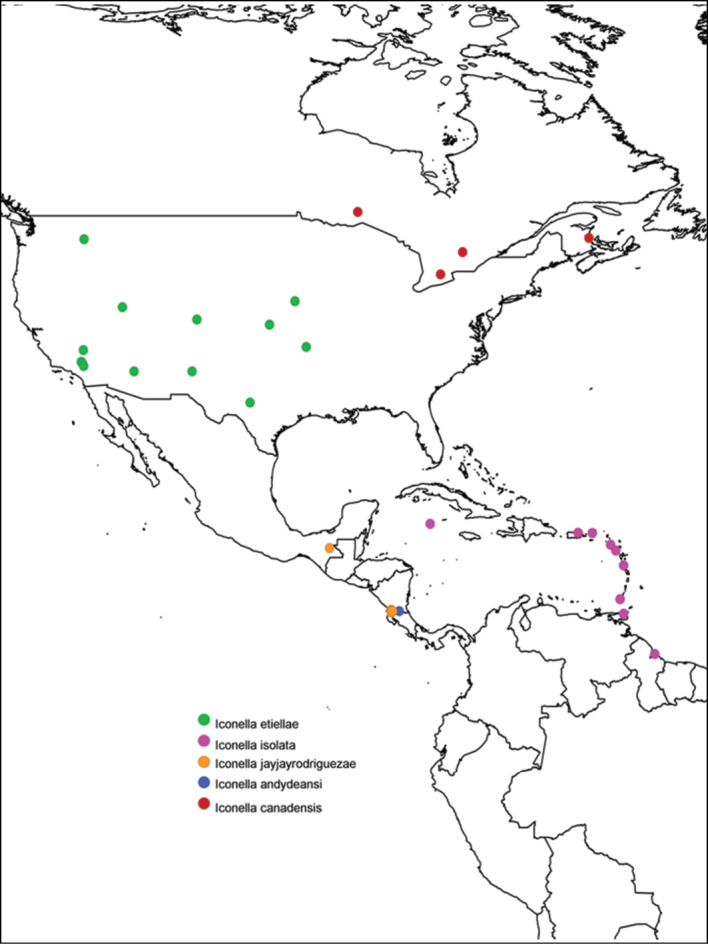
Distribution of New World species of *Iconella*. Species are individually color coded.

All of the described species have a different, well delimited geographic range (Fig. 3) and also differ in their known hosts. Most of the host species in the Western Hemisphere belong to the Lepidoptera families Pyralidae and Crambidae (with appropriate caution that what was called Pyralidae in the past is in part called Crambidae at present). The NJ tree (Fig. 2) as well as Bayesian (Fig. 1), ML, and parsimony based phylogenetic analyses of the barcodes support the validity of all New World species recognized herein (there is no molecular data available for *Iconella isolata*). However, the barcoding dataset did not contain a strong enough phylogenetic signal to resolve the phylogenetic relationships among *Iconella* species.

While these DNA barcodes and short COI fragments (mini-barcodes (Hajibabaei et al. 2006, Meusnier et al. 2008)) were sufficient to unambiguously separate the species of *Iconella*, more collections and longer DNA sequences will be needed to test the monophyly (or lack thereof) of *Iconella*.

### Key to the New World species of *Iconella*

**Table:** 

1	Propodeum mostly smooth and polished (as in Fig. 21), except for sparse punctures on the anterior 0.2 × of propodeum, and rather small carinae radiating from median longitudinal carina; metatibia mostly yellow, at most with very small and faint brown spot on posterior 0.1 or less, and metatarsus mostly yellow, except for brown area on posterior half of first tarsomerus (Figs 17, 19, 23, 25); fore wing with most veins transparent or white, vein margins of same color than interior of vein (Figs 18, 24)	2
–	Propodeum with anterior 0.2 × covered with punctures, posterior 0.8 covered with mix of punctures, striated sculpture and carinae (as in Fig. 15); metatibia with brown to black coloration on posterior 0.2–0.4, and metatarsus mostly dark brown, except for yellowish area on anterior half of first tarsomerus (as in Fig. 33); fore wing with at least some veins with thin brown margins and interior of veins yellow to light brown (Figs 5, 11, 31)	3
2	Pterostigma almost completely brown, with only small whitish spot anteriorly (Fig. 18); humeral complex half yellow, half brown; profemur almost completely dark brown (yellow area absent or limited to posterior 0.2); interocellar distance 2.4 × or more posterior ocellus diameter (Fig. 20); mediotergite 2 width at posterior margin 4.6 × or less its length (Fig. 22); larger species, body length (head to apex of metasoma) 3.0 mm or more, and fore wing length 3.3 mm or more. [Western and central United States: AR, AZ, CA, CO, IA, NM, OK, and WA. Hosts: *Etiella zinckenella*, *Olycella junctolineella*, *Psorosina hammondi*, and *Ufa rubedinella* (Pyralidae)]	*Iconella etiellae* (Viereck, 1911)
–	Pterostigma mostly transparent or whitish, with only thin brown margins (Fig. 24); humeral complex yellow to white; profemur mostly yellow, dark brown area limited to anterior 0.2 or less; interocellar distance 2.1 × or less posterior ocellus diameter; mediotergite 2 width at posterior margin 5.0 × or more its length (Fig. 26); smaller size, body length (head to apex of metasoma) 3.0 mm or less, and fore wing length 3.2 mm or less. [Caribbean islands and northern part of South America: British Virgin Islands, Cayman Islands, Dominica, Grenada, Guyana, Montserrat, Puerto Rico, Saint Kitts & Nevis, Trinidad & Tobago. Host: *Ancylostomia stercorea* (Pyralidae)]	*Iconella isolata* (Muesebeck, 1955), stat. r.
3-	Ocular-ocellar line 1.6 × posterior ocellus diameter; humeral complex half yellow, half brown; mediotergite 1 width at anterior margin 2.2 × or less its width at posterior margin (Fig. 13); ovipositor sheaths length 0.8 × or less metatibial length (Fig. 12); larger species, body length (head to apex of metasoma) 3.5 mm or more (rarely 3.2 mm) and fore wing length 3.5 mm or more; an extra-tropical species distributed in North America north of 40° N (Canada). [Eastern Canada: NB, ON, and QC. Host: *Epinotia solandriana* (Tortricidae) and, likely, *Acrobasis betulella* (Pyralidae)]	*Iconella canadensis* Fernández-Triana, sp. n.
-	Ocular-ocellar line 2.0 × or more posterior ocellus diameter; humeral complex fully yellow to white; mediotergite 1 width at anterior margin 3.1 × or more its width at posterior margin (Figs 9, 36); ovipositor sheaths length 1.1 × metatibial length (Figs 6, 32); smaller size, body length (head to apex of metasoma) 3.0 mm or less, and fore wing length 3.3 mm or less; tropical species from Central America south of 17° N (Mexico and Costa Rica)	4
4	Profemur mostly yellow, dark brown area limited to anterior 0.2 or less; meso- and meta- femora mostly dark brown, with proximal 0.1–0.2 yellow to orange; mesoscutellar disc sculpture centrally smooth with few, scattered punctures near margins (Fig. 36); mediotergite 2 width at posterior margin 4.1 × or less its maximum length medially (Fig. 36); body length (head to apex of metasoma) 2.9–3.0 mm; fore wing length 3.2–3.3 mm. [Costa Rica (ACG) and Mexico (Chiapas). Host: undescribed species of Phycitinae (Pyralidae)]	*Iconella jayjayrodriguezae* Fernández-Triana, sp. n.
–	Profemur dark brown on anterior half, yellow on posterior half; meso- and meta- femora usually fully dark brown to black; mesoscutellar disc sculpture mostly with punctures scattered all over disc surface (Fig. 9); mediotergite 2 width at posterior margin 4.4 × its maximum length medially (Fig. 9); body length (head to apex of metasoma) 2.7–2.8 mm; fore wing length 3.0 mm. [Costa Rica (ACG). Host: undescribed species of Spilomelinae (Crambidae)]	*Iconella andydeansi* Fernández-Triana, sp. n.

## Taxonomic treatment of species, in alphabetical order

### Iconella andydeansi

Fernández-Triana sp. n.

http://zoobank.org/C9173C9E-1E3C-46A8-A459-97DDF2ABF1DF

http://species-id.net/wiki/Iconella_andydeansi

Figures 4–9

#### Type locality.

COSTA RICA, Alajuela, Area de Conservacion Guanacaste, Sector Rincon Rain Forest, Camino Rio Francia, 410m. Lat: 10.90425, Long: -85.28651.

#### Holotype.

♀, CNC. First label: DHJPAR0043035. Second label: Voucher: D.H.Janzen & W.Hallwachs, DB: http://janzen.sas.upenn.edu, Area de Conservacion Guanacaste, COSTA RICA, 11-SRNP-41294. Collecting date of caterpillar host: 20.iii.2011, collection date (eclosion date) of wasp: 05.iv.2011.

#### Specimens examined.

Paratypes: 1 ♀, 1 ♂ (CNC) Costa Rica, same locality than holotype. Specimens voucher codes: DHJPAR0042995 and DHJPAR0043008.

#### Description.

**Figures 4–9.**
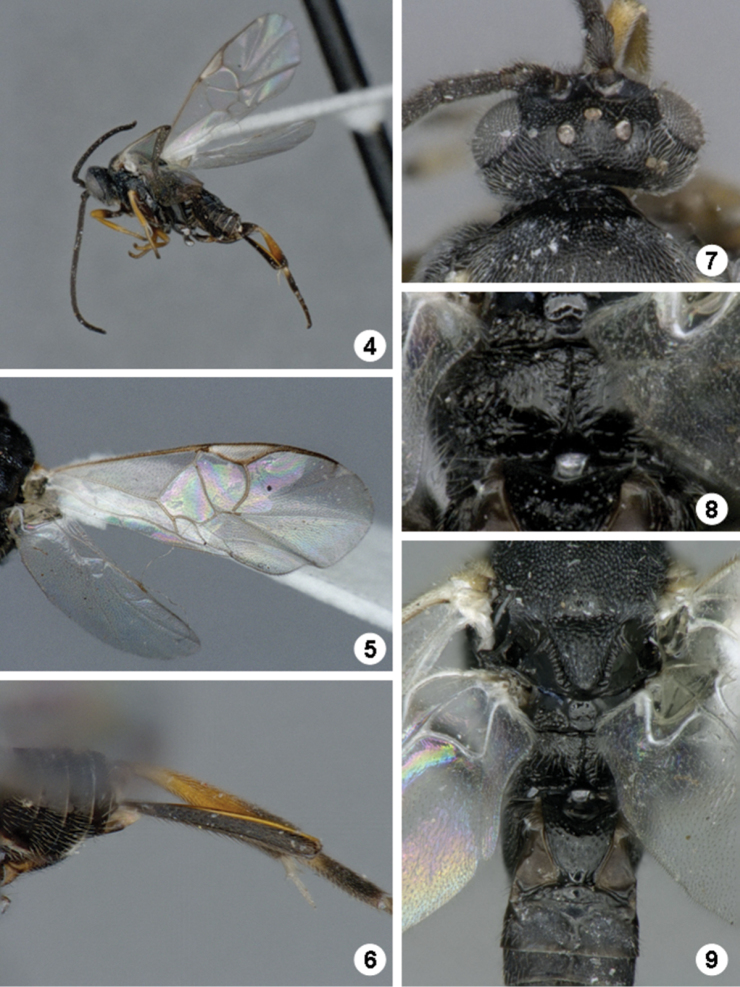
*Iconella andydeansi*. **4** Habitus, lateral view **5** Fore wing **6** Ovipositor sheats and metatibia **7** Head, dorsal view **8** Propodeum **9** Meso- and metasoma (partially), dorsal view.

*Promefur color*: dark brown on anterior half, yellow on posterior half. *Meso- and meta- femur color*: fully dark brown to black (Fig. 4). *Metatibia and metatarsus color*: Metatibia with brown to black coloration on posterior 0.2–0.4 ×; metatarsus mostly dark brown, except for yellowish area on anterior half of first tarsomerus (Fig. 6). *Tegula and humeral complex color*: tegula and humeral complex fully yellow to yellowish-white (Fig. 9). *Pterostigma color*: centrally yellow-white, with thin brown margins (Fig. 5). *Fore wing veins color*: at least some veins with thin brown margins and interior of veins yellow to light brown. *Body length (head to apex of metasoma)*: 2.7 mm or 2.8 mm. *Fore wing length*: 3.0 mm. *Ocular-ocellar line/posterior ocellus diameter*: 2.2 ×. *Interocellar distance/posterior ocellus diameter*: 2.1 × (Fig. 7). *Antennal flagellomere 2 length/width*: 2.5 × or 2.7 ×. *Antennal flagellomere 14 length/width*: 1.3 ×. *Length of flagellomere 2/length of flagellomere 14*: 2.4 ×. *Metafemur length/width*: 3.1 ×. *Mesoscutellar disc*: mostly with punctures scattered all over disc surface (Fig. 9). *Number of pits in scutoscutellar sulcus*: usually 12 or less, ocasionally reaching up to 14 pits. *Propodeum background sculpture*: anterior 0.2-0.4 × with rather dull puntures; posterior 0.6–0.8 × mostly sculptured, with mix of small puntures and carinae (mostly radiating from strong, longitudinal median carina) (Fig. 8). *Mediotergite 1 width at anterior margin/width at posterior margin*: 3.4 ×. *Mediotergite 2 width at posterior margin/length*: 4.4 × (Fig. 9). *Ovipositor sheaths length/metatibial length*: 1.1 × (Fig. 6).

**Male.** As female, although sculpture is slightly smoother.

#### Molecular data.

We analyzed three full 658 bp barcodes for this species.

#### Biology/ecology.

Host: An undescribed species of Phycitinae (Pyralidae) with provisional name “phyjanzen021 Janzen855” in the ACG database (http://janzen.sas.upenn.edu/caterpillars/database.lasso). Caterpillar collected while feeding on the foliage of *Lepidoploa salzmannii* (Asteraceae).

#### Distribution.

Only known from the holotype locality, in Sector Rincon Rain Forest of ACG at 410 m.

#### Comments.

The species has been reared only in one place from three caterpillars collected at the same time on the same species of food plant, out of 12,000+ rearings of ACG Pyralidae of more than 200 species. A single additional specimen, identified by DNA barcoding, has been reared in 2012 from the same species of host caterpillar in the same place and on the same food plant, but was not available for study. *Iconella andydeansi* is sympatric with *Iconella jayjayrodriguezae* in ACG, the latter being a species with a slightly larger ACG rain forest distribution but equally narrow food plant record, and also known from Chiapas, Mexico. Those are the only two species of New World *Iconella* that are known to be sympatric, and that was revealed through extensive collecting in ACG and the use of DNA barcodes. It is likely that further collecting in other areas of the Neotropics, as well as the barcoding of more fresh specimens, will reveal additional species.

#### Etymology.

This species is named in honor of Andy R. Deans (Pennsylvania State University, United States) in recognition of his major contribution to the taxonomy of the many species in the microgatrine genus *Alphomelon* that occur in Area de Conservación Guanacaste (e.g., Deans et al. 2003).

### Iconella canadensis

Fernández-Triana sp. n.

http://zoobank.org/C3E8D164-ABBC-4823-9D56-50E302065F55

http://species-id.net/wiki/Iconella_canadensis

Figures 10–16

#### Type locality.

CANADA. Ontario, Black Sturgeon Lake. Lat: 49.368333, Long: -88.881944.

#### Holotype.

♀, CNC. First label: Black Sturgeon Lake, Ontario, Em. 1-6-viii-1961, Insectary. Second label: Nest 97, Cell4ex provisions. Third label: W61319. Fourth label: Host either A. betullela or Rh. hasta. Fifth label: DNA Voucher CNCHYM 01498.

#### Specimens examined.

Paratypes: 3 ♀ (CNC) Canada: ON, Black Sturgeon Lake, 21–29.vii.1961, 26.vii.1962, and 2.viii.1962, ex: Provisions Nests 52 and 66, one specimen with DNA Voucher CNCHYM01497; 1 ♀ (CNC) Canada: ON, Galt, 11.vii.1952; 1 ♀ (CNC) Canada: ON, Whitney, 4.vii.1949, ex: Phalaenidae; 1 ♀, 1 ♂ (CNC) Canada: NB, Kouchibouguac National Park, 30.viii.1967, code-6060B, DNA Voucher CNCHYM01511 and CNCHYM01512; 1 ♀ (LFS) Canada: QC, Saint-Cléophas-de-Brandon, 4.vii.1968, ex: *Epinotia solandriana* on *Betula papyrifera*. Collecting dates of specimens examined: July and August (1949–1967).

#### Description.

**Figures 10–16.**
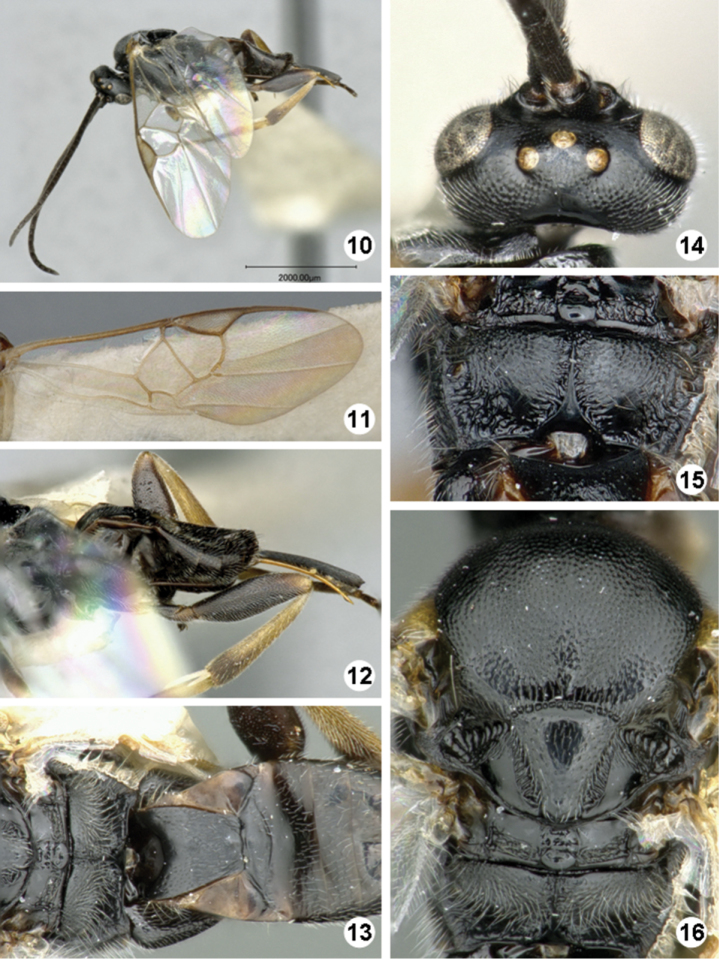
*Iconella canadensis*. **10** Habitus, lateral view **11** Fore wing **12** Ovipositor sheats, mesofermur, and metatibia **13** Propodeum, mediotergites 1–4, dorsal view **14** Head, dorsal view **15** Propodeum **16** Mesosoma, dorsal view.

*Promefur color*: dark brown on anterior half, yellow on posterior half. *Meso- and meta- femur color*: mostly dark brown but with proximal 0.1–0.2 × yellow to orange (Fig. 12). *Metatibia and metatarsus color*: Metatibia with brown to black coloration on posterior 0.2–0.4 ×; metatarsus mostly dark brown, except for yellowish area on anterior half of first tarsomerus (Fig. 10). *Tegula and humeral complex color*: tegula and anterior half of humeral complex yellow to yellowish-white, posterior half of humeral complex light brown to dark brown. *Pterostigma color*: centrally yellow-white, with thin brown margins, rarely mostly brown, with yellowish-white spot at anterior 0.2 × (Fig. 11). *Fore wing veins color*: at least some veins with thin brown margins and interior of veins yellow to light brown. *Body length (head to apex of metasoma)*: 3.5 mm, 3.6 mm, 3.7 mm, rarely 3.2 mm. *Fore wing length*: 3.8 mm, 3.9 mm, 4.0 mm, 4.1 mm or 4.2 mm. *Ocular-ocellar line/posterior ocellus diameter*: 1.6 ×. *Interocellar distance/posterior ocellus diameter*: 1.9 × (Fig. 14). *Antennal flagellomere 2 length/width*: 3.0 ×. *Antennal flagellomere 14 length/width*: 1.6 ×. *Length of flagellomere 2/length of flagellomere 14*: 2.1 ×. *Metafemur length/width*: 3.2 ×, 3.3 ×, rarely 3.4 ×. *Mesoscutellar disc*: mostly smooth with few, scattered punctures near margins (Fig. 16). *Number of pits in scutoscutellar sulcus*: usually 16, ocasionally only 14 pits. *Propodeum background sculpture*: anterior 0.2-0.4 × with rather dull puntures; posterior 0.6-0.8 × mostly sculptured, with mix of small puntures and carinae (mostly radiating from strong, longitudinal median carina) (Fig. 15). *Mediotergite 1 width at anterior margin/width at posterior margin*: 2.1 × or 2.2 ×. *Mediotergite 2 width at posterior margin/length*: 3.6 ×, 3.8 × or 4.4 × (Fig. 13). *Ovipositor sheaths length/metatibial length*: 0.7 × or 0.8 × (Fig. 12).

**Male.** As female.

#### Molecular data.

We analyzed three short 164 bp COI sequences from the DNA barcode region.

#### Biology/ecology.

Host: *Epinotia solandriana* (Tortricidae) and likely *Acrobasis betulella* (Pyralidae) (see Comments below).

#### Distribution.

Eastern Canada: NB, ON, QC.

#### Comments.

The holotype has a label stating that it emerged from either *Acrobasis betulella* (Pyralidae) or *Rheumaptera hasta* (Geometridae). Based on the known biology of the genus *Iconella* in the world, the second alternative is unlikely, and thus we consider *Acrobasis betulella* as the potential host in that case. However, the pyralid host cannot be taken as definitive until more reared specimens confirm the decision.

#### Etymology.

The name refers to the known distribution of the species, in Eastern Canada.

### Iconella etiellae

(Viereck, 1911)

http://species-id.net/wiki/Iconella_etiellae

Figures 17–22

Apanteles etiellae : Viereck 1911: 178.
Iconella etiellae (Viereck). Mason 1981: 75.

#### Type locality.

UNITED STATES, Washington, Pullman.

#### Holotype.

♂, NMNH (examined).

#### Specimens examined.

3 ♀, 3 ♂ (CNC) United States: CA (Apple Valley, Helendale, Kramer Hills and Panamint Valley) and UT (Dugway Proving Ground). Collecting dates of specimens examined: May and June (1953–1961).

#### Description.

**Figures 17–22.**
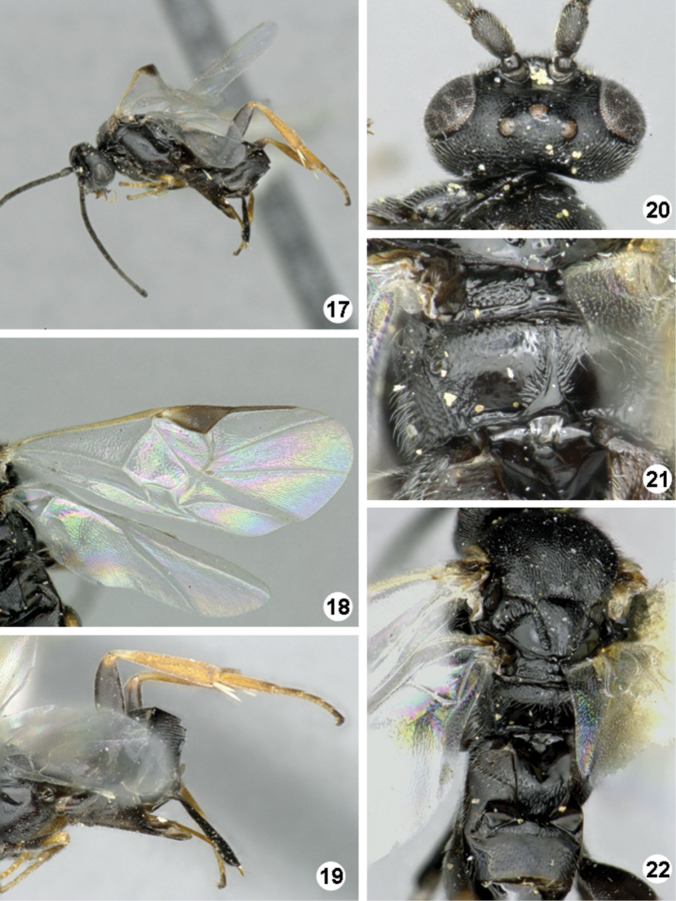
*Iconella etiellae*. **17** Habitus, lateral view **18** Fore wing **19** Ovipositor sheats, mesofermur, and metatibia **20** Head, dorsal view **21** Propodeum **22** Meso- and metasoma (partially), dorsal view.

*Promefur color*: almost completely dark brown (yellow area absent or limited to posterior 0.2 x) (Fig. 17). *Meso- and meta- femur color*: mostly dark brown but with proximal 0.1-0.2 × yellow to orange (Fig. 19). *Metatibia and metatarsus color*: Metatibia mostly yellow, at most with very small and faint brown spot on posterior 0.1 × or less; metatarsus mostly yellow, except for brown area on posterior half of first tarsomerus (Fig. 19). *Tegula and humeral complex color*: tegula and anterior half of humeral complex yellow to yellowish-white, posterior half of humeral complex light brown to dark brown. *Pterostigma color*: mostly brown, with yellowish-white spot at anterior 0.2 × (Fig. 18). *Fore wing veins color*: most of veins transparent or at most yellowish-white, margins of same color than interior of vein. *Body length (head to apex of metasoma)*: 3.0 mm or 3.1 mm. *Fore wing length*: 3.3 mm or 3.4 mm. *Ocular-ocellar line/posterior ocellus diameter*: 1.6 × or 1.7 ×. *Interocellar distance/posterior ocellus diameter*: 2.4 × or 2.6 × (Fig. 20). *Antennal flagellomere 2 length/width*: 2.6 × or 2.7 ×. *Antennal flagellomere 14 length/width*: 1.4 × or 1.7 ×. *Length of flagellomere 2/length of flagellomere 14*: 2.2 ×. *Metafemur length/width*: 3.2 × or 3.3 ×. *Mesoscutellar disc*: mostly smooth with few, scattered punctures near margins (Fig. 22). *Number of pits in scutoscutellar sulcus*: usually 14 or more, ocasionally reaching up to 16 pits. *Propodeum background sculpture*: anterior 0.2–0.4 × with fine puntures; posterior 0.6–0.8 × mostly smooth, at most with some small carinae (mostly radiating from strong, longitudinal median carina) (Fig. 21). *Mediotergite 1 width at anterior margin/width at posterior margin*: 2.4 × or 2.5 ×. *Mediotergite 2 width at posterior margin/length*: 5.0 × or 5.1 × (Fig. 22). *Ovipositor sheaths length/metatibial length*: 1.1 × (Fig. 19).

**Male.** As female.

#### Molecular data.

We analyzed one 607 bp barcode for this species.

#### Biology/ecology.

Hosts: *Etiella zinckenella*, *Olycella junctolineella*, *Psorosina hammondi*, and *Ufa rubedinella* (Pyralidae).

#### Distribution.

United States: AR, AZ, CA, CO, IA, NM, OK, WA.

#### Comments.

There is a record from Virginia, United States (Yu et al. 2012). While we have not been able to examine that specimen, based in the known distribution of New World species of *Iconella* and their hosts, it is very likely that the VA specimen actually belongs to *Iconella canadensis* Fernández-Triana.

### Iconella isolata

(Muesebeck, 1955) stat. r.

http://species-id.net/wiki/Iconella_isolata

Figures 23–29

Apanteles etiellae isolatus Muesebeck, 1955: 161.

#### Type locality.

TRINIDAD & TOBAGO, St. Augustine.

#### Holotype.

♀, NMNH (not examined). **Paratypes.** 2 ♀ and 1 ♂, CNC (examined).

#### Specimens examined.

11 ♀, 5 ♂ (CNC) British Guiana, Cayman islands (Grand Cayman, Georgetown), Grenada, Montserrat, Saint Kitts & Nevis (Nevis, Round Hill), Trinidad & Tobago (Paradise Mt., St. Augustine, and Tacarigua). Collecting dates of specimens examined: February, March and December (1950–1965).

#### Description.

*Promefur color*: mostly yellow, dark brown area limited to anterior 0.2 or less, rarely dark brown on anterior half, yellow on posterior half. *Meso- and meta- femur color*: mostly dark brown but with proximal 0.1–0.2 × yellow to orange. *Metatibia and metatarsus color*: Metatibia mostly yellow, at most with very small and faint brown spot on posterior 0.1 × or less; metatarsus mostly yellow, except for brown area on posterior half of first tarsomerus. *Tegula and humeral complex color*: tegula and humeral complex fully yellow to yellowish-white (Fig. 29). *Pterostigma color*: centrally transparent, with yellow-white margins (Fig. 24). *Fore wing veins color*: most of veins transparent or at most yellowish-white, margins of same color than interior of vein. *Body length (head to apex of metasoma)*: 2.4 mm, 2.5 mm, 2.6 mm, 2.7 mm, 2.8 mm or 2.9 mm. *Fore wing length*: 2.8 mm, 2.9 mm, 3.0 mm, 3.1 mm or 3.2 mm. *Ocular-ocellar line/posterior ocellus diameter*: 1.9 × or 2.0 ×. *Interocellar distance/posterior ocellus diameter*: 1.9 ×, 2.0 × or 2.1 × (Fig. 27). *Antennal flagellomere 2 length/width*: 2.2 ×, 2.3 × or 2.5 ×. *Antennal flagellomere 14 length/width*: 1.3 × or 1.4 ×. *Length of flagellomere 2/length of flagellomere 14*: 2.3 ×, 2.4 × or 2.5 ×. *Metafemur length/width*: 3.2 × or 3.3 ×. *Mesoscutellar disc*: mostly smooth with few, scattered punctures near margins (Fig. 29). *Number of pits in scutoscutellar sulcus*: usually 12 or less, ocasionally reaching up to 14 pits. *Propodeum background sculpture*: anterior 0.2–0.4 × with fine puntures; posterior 0.6–0.8 × mostly smooth, at most with some small carinae (mostly radiating from strong, longitudinal median carina) (Fig. 28). *Mediotergite 1 width at anterior margin/width at posterior margin*: 2.1 ×, 2.6 × or 2.8 ×. *Mediotergite 2 width at posterior margin/length*: 3.6 ×, 3.7 ×, 3.8 ×, 4.5 × or 4.6 × (Fig. 26). *Ovipositor sheaths length/metatibial length*: 1.0 × (Fig. 25).

**Male.** As female.

**Figures 23–29.**
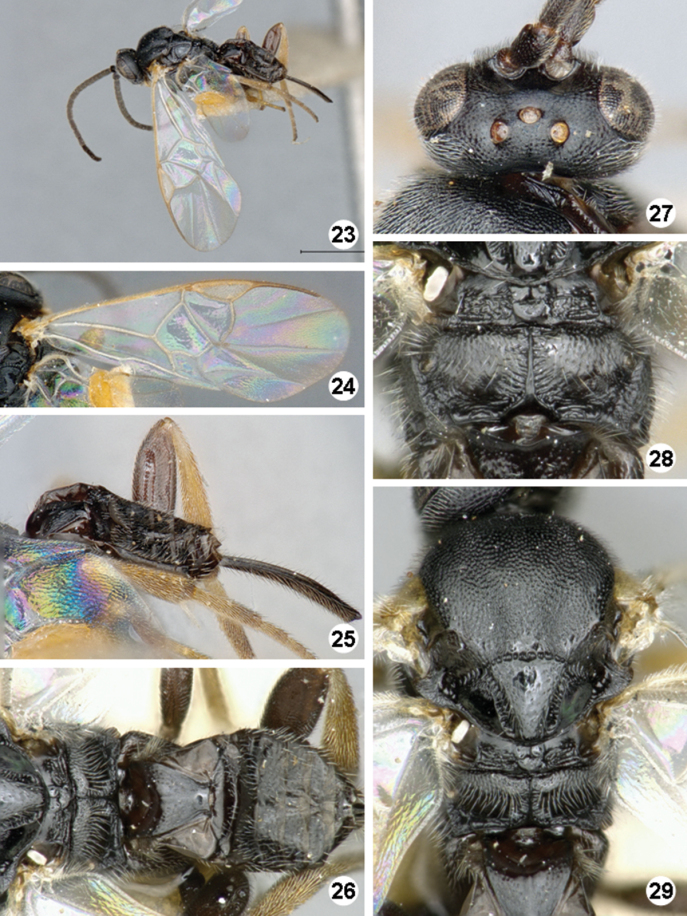
*Iconella isolata*. **23** Habitus, dorso-lateral view **24** Fore wing **25** Ovipositor sheats, mesofermur, and metatibia **26** Propodeum and metasoma, dorsal view **27** Head, dorsal view **28** Propodeum **29** Mesosoma and mediotergite **1** dorsal view.

#### Molecular data.

No DNA barcode sequence was available for this species, the only one among the New World species without molecular data.

#### Biology/ecology.

Host: *Ancylostomia stercorea* (Pyralidae).

#### Distribution.

British Virgin Islands, Cayman Islands, Dominica, Grenada, Guyana, Montserrat, Puerto Rico, Saint Kitts & Nevis, Trinidad & Tobago.

#### Comments.

Until now, *Iconella isolata* had been considered to be a subspecies of *Iconella etiellae*. After study of numerous specimens, we have found consistent and significant differences in morphology, hosts and geographical distribution and thus consider *Iconella isolata* as a different species on its own. Cayman Islands and Saint Kitts & Nevis are new locality records, based on CNC specimens. The specimen from Cayman Islands represents the westernmost locality, and it expands considerably the previously known distribution of the species in the Caribbean islands.

### Iconella jayjayrodriguezae

Fernández-Triana sp. n.

http://zoobank.org/A1E2E3FC-6C2D-4E6F-83C0-AD388D55DCB5

http://species-id.net/wiki/Iconella_jayjayrodriguezae

Figure 30–36

#### Type locality.

COSTA RICA, Alajuela, Area de Conservacion Guanacaste, Sector Rincon Rain Forest, Sendero Venado, 420m. Lat: 10.89678, Long: -85.27001.

#### Holotype.

♀, NMNH. First label: DHJPAR0039740. Second label: Voucher: D.H.Janzen & W.Hallwachs, DB: http://janzen.sas.upenn.edu, Area de Conservacion Guanacaste, COSTA RICA, 09-SRNP-41791. Collecting date of caterpillar host 21.vii.2009, collection date (eclosion date) of wasp 10.viii.2009.

#### Specimens examined.

Paratypes: 3 ♀, 1 ♂ (CNC) Costa Rica, Alajuela, ACG, Sector San Cristobal, Rio Blanco Abajo, 500m, Lat: 10.90037  Long: -85.37254; 1 ♀ (CNC) Mexico, Chiapas, 16°58'N, 91°47'W, 23–25.viii.1978. Collecting dates of specimens examined: January, March, April, June, July, and September (2009-2011) for Costa Rican specimens; August (1978) for Mexican specimen.

#### Description.

*Promefur color*: mostly yellow, dark brown area limited to anterior 0.2 or less. *Meso- and meta- femur color*: mostly dark brown but with proximal 0.1–0.2 × yellow to orange. *Metatibia and metatarsus color*: Metatibia with brown to black coloration on posterior 0.2–0.4 ×; metatarsus mostly dark brown, except for yellowish area on anterior half of first tarsomerus (Fig. 33). *Tegula and humeral complex color*: tegula and humeral complex fully yellow to yellowish-white. *Pterostigma color*: centrally transparent, with yellow-white margins, rarely centrally yellow-white, with thin brown margins (Fig. 31). *Fore wing veins color*: at least some veins with thin brown margins and interior of veins yellow to light brown. *Body length (head to apex of metasoma)*: 2.9 mm or 3.0 mm. *Fore wing length*: 3.2 mm or 3.3 mm. *Ocular-ocellar line/posterior ocellus diameter*: 2.0 ×. *Interocellar distance/posterior ocellus diameter*: 1.9 × (Fig. 34). *Antennal flagellomere 2 length/width*: 2.3 ×, 2.4 × or 2.5 ×. *Antennal flagellomere 14 length/width*: 1.3 ×, rarely 1.1 × or 1.5 ×. *Length of flagellomere 2/length of flagellomere 14*: 2.3 ×, 2.4 ×, 2.5 × or 2.6 ×. *Metafemur length/width*: 3.2 ×, 3.3 × or 3.4 ×. *Mesoscutellar disc*: mostly smooth with few, scattered punctures near margins (Fig. 36). *Number of pits in scutoscutellar sulcus*: usually 12 or less, ocasionally reaching up to 14 pits. *Propodeum background sculpture*: anterior 0.2–0.4 × with rather dull puntures; posterior 0.6–0.8 × mostly sculptured, with mix of small puntures and carinae (mostly radiating from strong, longitudinal median carina) (Fig. 35). *Mediotergite 1 width at anterior margin/width at posterior margin*: 3.1 ×, 3.2 × or 3.3 ×. *Mediotergite 2 width at posterior margin/length*: 3.7 ×, 3.8 ×, 3.9 × or 4.1 × (Fig. 36). *Ovipositor sheaths length/metatibial length*: 1.1 × (Fig. 32).

**Male.** As female.

**Figures 30–36.**
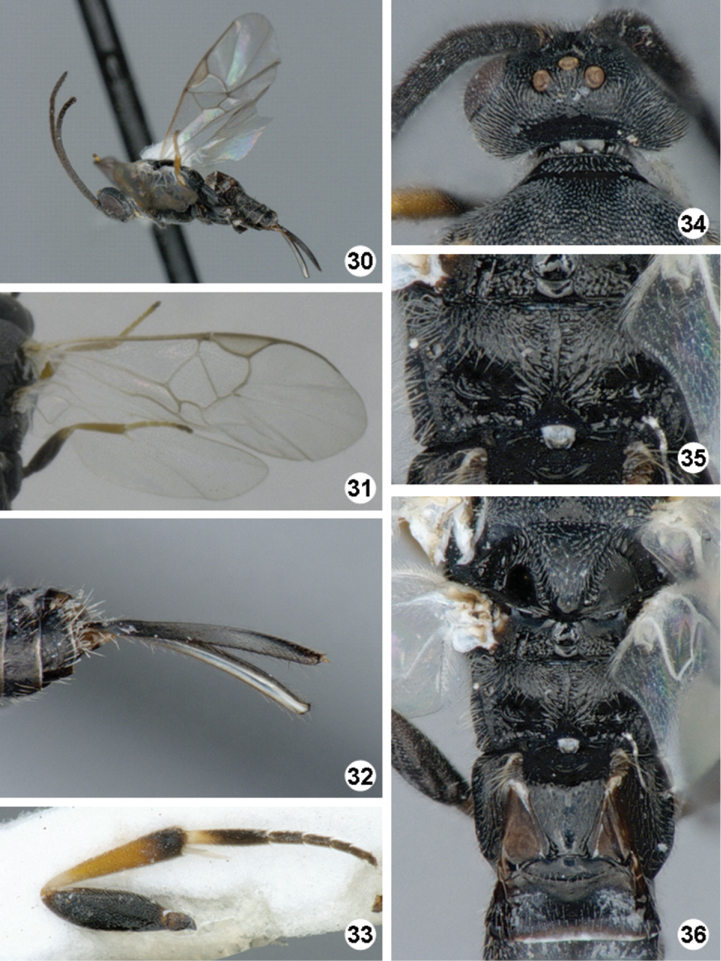
*Iconella jayjayrodriguezae*. **30** Habitus, lateral view **31** Fore wing **32** Ovipositor sheaths **33** Hind leg **34** Head, dorsal view **35** Propodeum **36** Meso- and metasoma (partially), dorsal view.

#### Molecular data.

We analyzed eight 650–658 bp barcodes for this species, one from Mexico and seven from Costa Rica.

#### Biology/ecology.

Host: An undescribed species of Spilomelinae (Crambidae) with provisional name “spiloBioLep01 BioLep414” in the ACG database (http://janzen.sas.upenn.edu/caterpillars/database.lasso). Caterpillar collected while feeding on the foliage of *Lepidoploa salzmannii* and *Lepidoploa tortuosa* (Asteraceae).

#### Distribution.

Costa Rica (ACG), and Mexico (Chiapas). In ACG it has been reared from four rainforest localities between 420–500m, 2-10 km apart, in six different months.

#### Comments.

Although morphologically similar to *Iconella andydeansi*, *Iconella jayjayrodriguezae* is known from two very widely separated places in Central America, and the barcoding sequences of the two species differ by 8.7% (57 bp).

#### Etymology.

This species is named in honor of Josephine J. Rodriguez (National Center for Ecological Analysis and Synthesis, University of California, Santa Barbara, United States) in recognition of her outstanding enthusiasm for studying the taxonomy and biology of the microgastrine wasps of ACG (e.g., Smith et al. 2008).

## Supplementary Material

XML Treatment for Iconella andydeansi

XML Treatment for Iconella canadensis

XML Treatment for Iconella etiellae

XML Treatment for Iconella isolata

XML Treatment for Iconella jayjayrodriguezae

## Appendix

Lucid key to the New World species of the parasitoid wasp *Iconella* (Hymenoptera, Braconidae, Microgastrinae). (doi: 10.3897/zookeys.321.5160.app) File format: Lucid Key Data (lk4).
