# Supplementary material for: A review of the New World species of the parasitoid wasp Iconella (Hymenoptera, Braconidae, Microgastrinae)
Source: Zookeys. 2013 Aug 7;(321):65–87. doi: 10.3897/zookeys.321.5160 (PMC3744146; doi:10.3897/zookeys.321.5160)
Supplement: Supplementary file 6 — Lucid key to the New World species of the parasitoid wasp Iconella (Hymenoptera, Braconidae, Microgastrinae). (doi: 10.3897/zookeys.321.5160.app) File format: Lucid Key Data (lk4). [file ZooKeys-321-065-s001.zip › Iconella/Media/Html/desc_Apanteles_balthazari.html]

Natural Language Description


## Apanteles balthazari

COLOUR: Body color (head, meso and metasoma) Mostly dark brown to black (except for some sternites which may be pale). COLOUR: Antenna color Scape, pedicel and flagellum dark brown to black. COLOUR: Palpi color Pale. COLOUR: Coxae color (leg 1, 2, 3) Dark, dark, dark. COLOUR: Femur color (leg 1, 2, 3) Pale, pale, pale. COLOUR: Tibiae color (Pro, meso and metatibia) Pale, pale, pale. COLOUR: Tegula and wing base color Both pale. COLOUR: Pterostigma color Pale with only borders brown. COLOUR: Wing veins color Mostly white color or fully transparent. GENERAL: Body lenght (head to metasoma) 3.0-3.2 mm. GENERAL: Forewing length 3.0-3.2 mm. LEGS: Tarsal claws Simple. LEGS: Metacoxae sculpture Partially sculptured, with punctures especially on outer side. MESOSOMA: Mesoscutum punctures Deep, close punctures (separated by less than 2x its maximum diameter). MESOSOMA: Scutellum punctures Mostly smooth. MESOSOMA: Number of impressions in scutellar suture 7-8. MESOSOMA: Maximum height of smooth area on lateral face of scutellum 60-80%. MESOSOMA: Maximum width of smooth area on lateral face of scutellum <0.5x (or less) lateral face lenght. MESOSOMA: Definition of dorsal (anterior) and horizontal (posterior) parts of propodeum Anterior and posterior parts clearly marked by angulation of propodeum. MESOSOMA: Propodeum areola Complete, including trasverse carina reaching the spiracle. MESOSOMA: Propodeum background sculpture Mostly sculptured. METASOMA: Tergite 1 shape Parallel-sided or barrel shaped. METASOMA: Tergite 1 sculpture Diverse sculpture patterns, sometimes with medial excavated area with transverse striation and/or a polished knob centrally on the apical edge of tergite. METASOMA: Tergite 2 sculpture More or less fully sculptured with longitudinal striation. METASOMA: Hypopygium medial edge Medially strongly desclerotized and with many pleats. METASOMA: Hypopygium tip shape Angulated and/or poointed. METASOMA: Hypopygium size About the same size of last sternites. METASOMA: Ovipositor shape Evenly tapered. METASOMA: Ovipositor sheaths lenght 1.1-1.4x metatibial length. WINGS: Point of insertion of vein r in petrostigma Beyond the middle length of pterostigma. WINGS: Angulation of vein r regarding wing anterior margin Vein r slightly outwards (wing apex). WINGS: Veins r and 2RS shape Strongly angulated, sometimes with a knob.
