# Supplementary material for: A review of the New World species of the parasitoid wasp Iconella (Hymenoptera, Braconidae, Microgastrinae)
Source: Zookeys. 2013 Aug 7;(321):65–87. doi: 10.3897/zookeys.321.5160 (PMC3744146; doi:10.3897/zookeys.321.5160)
Supplement: Supplementary file 6 — Lucid key to the New World species of the parasitoid wasp Iconella (Hymenoptera, Braconidae, Microgastrinae). (doi: 10.3897/zookeys.321.5160.app) File format: Lucid Key Data (lk4). [file ZooKeys-321-065-s001.zip › Iconella/Media/Html/desc_Apanteles_rhomboidalis.html]

Natural Language Description


## Apanteles rhomboidalis

COLOUR: Body color (head, meso and metasoma) Mostly dark brown to black (except for some sternites which may be pale). COLOUR: Antenna color Scape, pedicel and flagellum pale. COLOUR: Palpi color Pale. COLOUR: Coxae color (leg 1, 2, 3) Pale, pale, partially pale/partially dark (extension of each color may vary). COLOUR: Femur color (leg 1, 2, 3) Pale, pale, pale. COLOUR: Tibiae color (Pro, meso and metatibia) Pale, pale, basally pale/apically dark (extension of each color may vary). COLOUR: Tegula and wing base color Both pale. COLOUR: Pterostigma color Brown, with small central pale area. COLOUR: Wing veins color Mostly brown (few veins may be unpigmented). GENERAL: Body lenght (head to metasoma) 2.1-2.3 mm. GENERAL: Forewing length 2.1-2.3 mm. LEGS: Tarsal claws Simple. LEGS: Metacoxae sculpture Partially sculptured, with punctures especially on outer side. MESOSOMA: Mesoscutum punctures Mostly shallow, dense punctures (separated by less than 2x its maximum diameter). MESOSOMA: Scutellum punctures Mostly smooth. MESOSOMA: Number of impressions in scutellar suture 11-12. MESOSOMA: Maximum height of smooth area on lateral face of scutellum 20%-50%. MESOSOMA: Maximum width of smooth area on lateral face of scutellum 0.6-0.7x lateral face width. MESOSOMA: Definition of dorsal (anterior) and horizontal (posterior) parts of propodeum Dorsal part greatly shortened but still defined, if less angulated. MESOSOMA: Propodeum areola More or less complete but with only partial or absent transverse carina. MESOSOMA: Propodeum background sculpture Mostly sculptured. METASOMA: Tergite 1 shape Mostly paralell-sided, but suddenly narrowing apically (1/2-1/4 of apex) so that basal width > apical width. METASOMA: Tergite 1 sculpture More or less fully sculptured with longitudinal striation. METASOMA: Tergite 2 sculpture More or less fully sculptured with longitudinal striation. METASOMA: Hypopygium medial edge Medially strongly desclerotized and with many pleats. METASOMA: Hypopygium tip shape Angulated and/or poointed (?). METASOMA: Hypopygium size Larger than last sternites (?). METASOMA: Ovipositor shape Evenly tapered. METASOMA: Ovipositor sheaths lenght 1.1-1.4x metatibial length. WINGS: Point of insertion of vein r in petrostigma Beyond the middle length of pterostigma. WINGS: Angulation of vein r regarding wing anterior margin Vein r more or less perpendicular to wing margin. WINGS: Veins r and 2RS shape Distinctly but not strongly angulated.
