# Supplementary figures and images for: A review of the New World species of the parasitoid wasp Iconella (Hymenoptera, Braconidae, Microgastrinae)
Source: Zookeys. 2013 Aug 7;(321):65–87. doi: 10.3897/zookeys.321.5160 (PMC3744146; doi:10.3897/zookeys.321.5160)

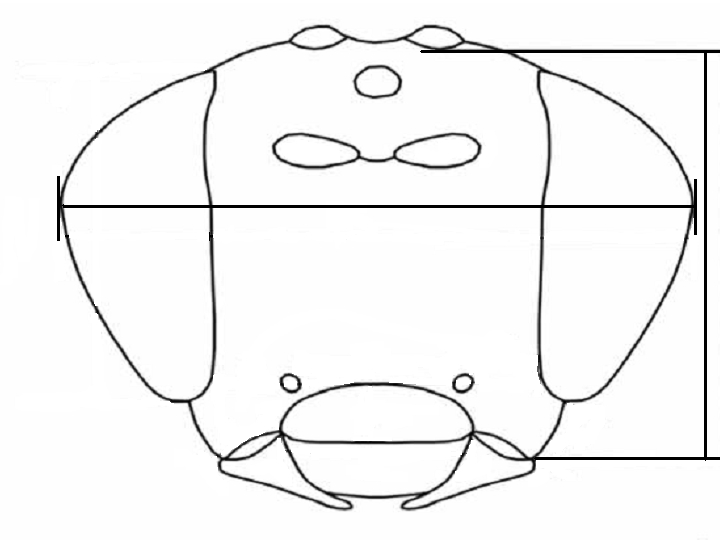

Supplement: Supplementary file 6 — Lucid key to the New World species of the parasitoid wasp Iconella (Hymenoptera, Braconidae, Microgastrinae). (doi: 10.3897/zookeys.321.5160.app) File format: Lucid Key Data (lk4). [file ZooKeys-321-065-s001.zip › Iconella/Media/Images/Head Measurements Template.jpg]

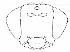

Supplement: Supplementary file 6 — Lucid key to the New World species of the parasitoid wasp Iconella (Hymenoptera, Braconidae, Microgastrinae). (doi: 10.3897/zookeys.321.5160.app) File format: Lucid Key Data (lk4). [file ZooKeys-321-065-s001.zip › Iconella/Media/Thumbs/Head Measurements Template_TN.jpg]
